# Small-Molecule Drugs in Pediatric Neuro-Oncology

**DOI:** 10.3390/curroncol32080417

**Published:** 2025-07-25

**Authors:** Stephanie Vairy, George Michaiel

**Affiliations:** 1 Division of Hematology-Oncology, Department of Pediatrics, Centre Hospitalier Universitaire de Sherbrooke, Université de Sherbrooke, Sherbrooke, QC J1H 5N4, Canada; 2 Centre de Recherche du CHUS, Sherbrooke, QC J1H 5N4, Canada; 3 Division of Hematology-Oncology, Department of Pediatrics, BC Children’s Hospital, University of British Colombia, Vancouver, BC V6H 3N1, Canada; george.michaiel@cw.bc.ca; 4 British Colombia Children’s Hospital Research Institute, Vancouver, BC V5Z 4h4, Canada

**Keywords:** pediatric neuro-oncology, targeted therapy, brain tumor

## Abstract

Advances in molecular diagnostics have enabled precision medicine approaches in pediatric neuro-oncology, with small-molecule drugs emerging as promising therapeutic candidates targeting specific genetic and epigenetic alterations in central nervous system (CNS) tumors. This review provides a focused overview of several small-molecule agents under investigation or in early clinical use, including ONC201, tazemetostat, vorasidenib, CDK inhibitors, selinexor, and aurora kinase A inhibitors, among others. Highlighted are their mechanisms of action, pharmacokinetic properties, early efficacy data, and tolerability in pediatric populations. Despite encouraging preclinical and early-phase results, most agents face limitations due to study heterogeneity, lack of large-scale pediatric randomized trials, and challenges in drug delivery to the CNS. The review underscores the critical need for robust prospective clinical trials for the integration of these therapies into pediatric neuro-oncology care.

## Simple Summary

Small-molecule therapies are emerging as promising tools in the treatment of pediatric brain tumors, particularly those driven by distinct genetic or epigenetic alterations. This review highlights several investigational agents that target key oncogenic pathways with varying degrees of early clinical activity. While many demonstrate CNS penetration and manageable toxicity, their roles remain uncertain due to limited pediatric-specific data and trial heterogeneity. Continued prospective research is critical to validate these agents and define their integration into frontline pediatric neuro-oncology care.

## 1. Introduction

As advanced molecular diagnostics have become more widely available in pediatric oncology, so has the opportunity to employ targeted therapies specific to genetic or molecular pathways activated within tumor cells. Small-molecule drugs are low-molecular-weight compounds that have the ability to enter cells and modulate biological processes through interactions with proteins, most often enzymes, receptors, or channels [1]. Several have been investigated in various pediatric central nervous system tumors or malignancies. Herein we provide a brief overview of select small molecular inhibitors, their current application, ongoing research, and future directions. The agents discussed below have been selected based on various factors, including molecules of special public interest and excitement (whether founded or unfounded), molecules with unique mechanisms of action/genetic modulation, novel therapies on the brink of further scientific exploration, and molecules in early development with the potential for implications across multiple disease types. While this will not be an exhaustive list, several other agents are also planned to be reviewed in this Special Issue.

## 2. Dordaviprone, Imipridone (ONC201, TIC10, NSC350625)

ONC-201 is a dopamine-receptor D2 (DRD2) antagonist and a caseinolytic protease P (ClpP) agonist. G protein-coupled receptors (GPCRs), such as DRD2, are a superfamily of membrane receptors and control several signaling pathways [2]. They have been associated with gliomagenesis via multiple mechanisms including the activation of EGFR, Ras, and angiogenesis pathways [3,4]. The overexpression of DRD2 has been demonstrated in CNS tumors, mostly gliomas, and when antagonized, it correlates with anti-cancer activity [4,5]. On the other hand, ClpP is an ATP-dependent mitochondrial protein that degrades mitochondrial respiratory chain proteins to disrupt energy homeostasis [6]. Several disturbances of the Krebs cycle are observed under the action of this drug.

H3K27M cells appear to use alpha-ketoglutarate (aKG) to maintain low methylation levels. In H3K27M-mutant DMG cell lines, ONC201 restores H3K27M trimethylation by blocking oxoglutarate dehydrogenase (OGDH) through the production of the aKG-derived isoform L-2-hydroxyglutarate (L-2HG) [7].

TNF-related apoptosis inducing ligand (TRAIL) is a strong and selective tumor suppressor that can induce tumor cell apoptosis independently of p53, which is often inactivated in cancer. Initially, in vivo and xenograft experiments on the TIC10 compound identified a mechanism of action through the upregulation of TRAIL via the inhibition of the Akt/MEK/ERK pathway. This leads to downstream Foxo3a dephosphorylation, translocation into the nucleus, upregulation of the TRAIL promoter, and thus TRAIL-mediated apoptosis with its pro-apoptotic death receptor DR5 [8,9].

It is a brain-penetrant molecule, administered orally. Its long half-life of 11.3 h was measured on an RP2D of 625 mg once every 3 weeks in the first clinical trial in adults [10]. Similar PK was observed in children with a half-life of about 8h [11].

In the first phase II study (NCT02525692) of oral ONC201 for adults with recurrent bevacizumab-naive glioblastoma, one 22-year-old patient enrolled with *H3K27M*-mutant thalamic glioma achieved a near-complete durable response [12]. Along with encouraging preclinical data on the susceptibility of glioma cells lines harboring the H3 K27M mutation to ONC201, this led investigators to turn their focus to studying this agent in patients with *H3K27M*-mutant diffuse midline glioma (DMG).

As known, the histone mutation H3K27M (including H3.3 and H3.1/H3.2) in DMG is associated with aggressive clinical behavior and a median overall survival (OS) of 11 to 15 months [13]. This mutation is most frequently identified in pediatric and young adult gliomas located in midline central nervous system (CNS) structures; due to the location of these tumors, surgical resection is limited, and no effective systemic therapy has been identified to date [14].

Since 2017, several reports have demonstrated the preliminary clinical efficacy of ONC201, as well as its high tolerability and ease of administration [7,15]. These have generated interest in patients and families around the world in search of hope, despite the lack of more concrete data.

A pediatric phase I trial in diffuse pontine glioma (DPG)/DMG established the RP2D at 625 mg orally weekly, scaled by body weight for children, demonstrated a very safe toxicity profile [11]. Among the side effects, nausea, vomiting, fatigue, headache, lymphopenia, and increased alanine aminotransferase were mostly grades 1–2 [11,15]. During this study, median progression-free survival (PFS) was 20.4 weeks for patients who were initiated on ONC201 after completing radiation but prior to tumor progression, with a median OS of 53.8 weeks. In six patients with recurrent tumors, the median PFS was 12.6 weeks. Five (22.7%) newly diagnosed patients who started ONC201 following radiation therapy but prior to tumor progression were alive at 2 years from diagnosis [11].

A study by Arrillaga-Romany et al. reports the results of 50 patients selected from clinical trials and expanded access programs (ONC006, ONC013, ONC014, ONC016) [15]. The overall response rate (ORR) was 20% with an OS of 13.7 months according to RANO-HGG. Patients in this study were mainly adults with relapsed/progressive DMG but excluding spinal and diffuse intrinsic pontine glioma (DIPG). 

Another publication by Venneti et al. pooled the results of a total of 71 patients from the ONC201-014 and ONC201-018 clinical trials and compared them with a historical cohort of 373 patients [7]. Median overall survival was 21.7 months from diagnosis when therapy was initiated prior to recurrence. This was statistically superior to the historical cohort whose median OS was 12 months. In patients who were initiated on ONC201 at the time of recurrence, the median OS was 9.2 months compared to 8.1 months for historical controls (non-significant).

A study was recently published by the French SFCE and describes 174 patients (including 102 children) with progressive H3K27M DMG who were treated with ONC201 on a compassionate access program [16]. Their median OS from diagnosis was 15.5 months and 4.7 months after ONC201 initiation. However, 37% also received a second course of radiotherapy.

The caveats of these studies are many and include dissimilar cohorts, drug initiation at variable timepoints, concurrent medication use including bevacizumab, and the selection of patients reported. Indeed, it is disappointing that now 8 years after the initial reports, no robust clinical trial has provided a clear assessment of the efficacy of this molecule. Unfortunately, the selection of patients reported and analyzed, the heterogeneity of the cohorts, and the inclusion of confounding factors such as concurrent medication use and radiation strongly limit the interpretation of the results.

The preliminary results of prospective trial ONC013 and ONC014 have recently been presented at an ASCO meeting [17]. The pediatric phase I trial ONC014 had an ORR of 9.1% (1 PR/11) in a population of recurrent H3K27M HGG, non-DIPG. The latter presented a 95% tumor reduction by RANO and a duration of response of 8.5 months. The safety profile was tolerable.

Studies are underway to try to further shed light on this therapy: PNOC022/NCT05009992 (combining either the PI3K/Akt inhibitor paxalisib or panobinostat with ONC201), BIOMEDE 2/NCT05476939 (randomizing against everolimus), and the international randomized placebo-controlled phase III ACTION/NCT05580562, which excludes diffuse pontine glioma (Table 1).

## 3. Epigenetic Modulators

Tazemetostat (EPZ-6438, Tazverik) is an oral selective inhibitor the of enhancer of zeste homolog 2 (EZH2). It competes with the S-adenosylmethionine (SAM) cofactor to inhibit EZH2, reducing the levels of the trimethylated lysine 27 of histone 3 (H3K27me3). 

EZH2 is the catalytic subunit of the polycomb repressive complex 2 (PRC2), an epigenetic regulator that catalyzes the mono-, di-, and trimethylation of lysine 27 of histone H3 (H3K27me3), leading to transcriptional repression [18].

Tazemetostat is orally bioavailable, rapidly absorbed with a short half-life, and not influenced by co-administration with food and gastric acid-reducing agents [19]. It is metabolized by CYP3A in the liver into three major inactive metabolites (M1, M3, M5) It is highly distributed in tissues but with a potential limited access to the central nervous system [20].

Tazemetostat is the first EZH2 inhibitor approved by the FDA; however, its indications are for patients aged 16 years and older with epithelioid sarcoma or relapsed/refractory EZH2-mutated follicular lymphoma [21].

The Children’s Oncology Group (COG) conducted a phase II study using tazemetostat for patients aged 1–21 with tumors harboring the loss of SMARCB1/SMARCA4 or EZH2 mutations (MATCH APEC1621C) [22]. Twenty patients with refractory solid tumors, brain tumors, lymphomas, and histiocytic disorders were enrolled. These included eight atypical teratoid rhabdoid tumors (ATRTs) and four malignant rhabdoid tumors. Only one objective response of 91% in two-dimensional size was observed on a patient who was Rosai-Dorfman SMARCA4-deficient and who received the drug during 26 cycles of 28 days (1200 mg/m^2^/dose BID). Four patients with SMARCB1 loss experienced stable disease as their best response. Among them, one patient with SMARCB1-deficient epithelioid sarcoma received the drug for 26 cycles and one patient with ATRT received it during nine cycles. This led to a 6-month PFS of 35% and a 6-month OS of 45%, suggesting a potential effect of tazemetostat on disease stabilization. The drug was well tolerated in this population, with a few grade 3 (7–14%) and no grade 4 or 5 results. 

This study highlighted the moderate activity of tazemetostat in the pediatric population, particularly in ATRT with a loss of SMARCB1/INI1. Given the number of patients experiencing stable disease, this treatment could potentially be more beneficial in combination. For example, it may be that EZH2 inhibitors stimulate production of the SASP chemokines CCL2 and CXCL9/10, leading to the infiltration of NK and T cells in mouse models and thus potentiating immunotherapy in cold immune environments [23].

The same EZH2 inhibition also sensitized IDH1 R132H-mutant glioma murine models to the HDAC inhibitor panobinostat [24].

There is also the question of using tazemetostat in patients with rhabdoid tumor predisposition syndrome (RTPS). These patients have a germline mutation in the epigenetic regulatory genes SMARCB1 (85–95%) or SMARCA4 (5–15%) [25,26]. As the SMARCB1 or SMARC4 mutation results in the uninhibited activity of polycomb repressor complex 2 (PRC2), including EZH2, the possibility of using EZH2 inhibitors such as tazemetostat appears attractive, either as an initial combination or for maintenance therapy. It remains a hypothesis to be proved and tested in a clinical trial.

However, the reported incidence of secondary hematological cancers such as myelodysplastic syndrome, acute myeloid leukemia, and B and T lymphoblastic leukemia/lymphoma in 1.7% of adult users is a major deterrent [27]. One pediatric case of T-LBL has been reported during a phase I trial [22]. Overall, these reports of hematological cancers occurring during or shortly after treatment with EZH2 inhibitors certainly raise concerns about their use and development. It may also imply that EZH2 inhibitors could trigger leukemia through their mechanism of action via the stimulation of hematopoietic progenitors [28].

In a recent meeting, the results of a phase I of valemetostat tosylate, a first-in-class dual inhibitor targeting EZH1 and EZH2, have been presented [29]. The population consisted of relapsed and refractory patients with solid or CNS disease between 3 and 19 years old. INI1 was negative in 43% of these patients. They demonstrated safety and RP2D at 250 mg/1.7 m^2^. However, one patient developed acute lymphocytic leukemia, another presented pneumocystis pneumonia, and two had pneumonitis. An objective response was observed in ATRT patients (2/3) and in 2/6 patients with INI1-negative tumors. Also, long-term control exceeding 1 year was noted in one glioma, two chordoma, one ATRT, and three neuroblastoma patients, leading to a 1-year OS of 60% and 1-year PFS of 26.7%. 

Panobinostat is a pan-histone deacetylase (HDAC) inhibitor. This inhibition results in increased histone acetylation, leading to an increased transcription of pro-differentiation genes. This would logically lead to the differentiation of SMARCB1-deficient tumor cells. Preclinical studies have demonstrated this differentiation notably in the orthopedic xenograft models of ATRT [30]. On the other hand, clinically, the drug seems difficult to tolerate for long-term use, with myelosuppression, increased ALT, nausea, and diarrhea reported even when taken intermittently for 3 days/week [31]. However, the pediatric study using panobinostat as maintenance in patients with ATRT (NCT04897880) had to be terminated early due to the withdrawal of the compound from the US market.

On another note, although panobinostat appeared to be very active in pre-clinical studies against DIPG [32,33], a phase I study by Monje et al. showed no significant efficacy but with an MTD of 10 mg/m^2^ [31]. It is not known whether the results could have been different had a different administration schedule been used, given the difficulty of reaching higher doses. Also, several preclinical studies have demonstrated a limited CNS penetration of panobinostat [34], although Homan et al. demonstrated an effective concentration in a mouse model [35].

Other HDACs, such as vorinostat, romidepsin, and belinostat, have been developed and are FDA-approved for hematological cancers. Vorinostat appears to have good blood–brain barrier (BBB) penetrance in the clinic, but less than 10-fold is needed, as demonstrated in pre-clinical models of group 3 medulloblastoma [34,36]. Moreover, Milde et al. demonstrated in a cell line model that HDACis such as vorinostat and panobinostat had a radiosensitizing effect on medulloblastoma cells and induced apoptosis [36]. More specifically, MYC-driven group 3 medulloblastoma cells have been shown to be highly susceptible to inhibition by HDACs and HDAC2 is highly expressed in group 3 medulloblastomas [37]. Ecker and colleagues have demonstrated that HDAC2 is located in a complex with most of the MYC-DNA binding sites, indicating a strong dependence of MYC on HDAC2 binding [38]. The specific targeting of MYC as an anti-cancer therapy remains challenging because of its complex network of interactions, its protein structure, short half-life, and numerous tumorigenic pathways involved; combination therapy may be a possible solution to overcome this problem, opening the door to new therapeutic ideas for this subgroup of patients. The concept of MYC/mTOR dual targeting has been well reviewed by Kumar et al., and the pre-clinical synergistic anti-tumor effect on MYC-driven medulloblastoma cells has been observed [39,40]. Panobinostat has also demonstrated an anti-tumor effect when combined with BET inhibitor JQ1 in MYC-driven medulloblastoma cell lines and xenografts [41]. 

While not tested yet, spinal ependymoma with MYCN amplification (SPE-MYCN) may benefit from these experimental approaches. Conversely, posterior fossa A (PFA) ependymoma are characterized by the oncogenic driver EZH inhibitory protein (EZHIP) and have the most dismal prognosis of all ependymoma subgroups [42]. As tumorigenesis appears similar to H3K27M-mutant DMG, it is reasonable to suppose that drugs designed for the latter, such as panobinostat, may be effective [32].

Corin is a dual-warhead molecule containing an HDAC1/2 inhibitor and an LSD1 inhibitor. It selectively inhibits the CoREST complex, an epigenetic repressor. It can therefore antagonize residual SWI/SNF activity in ATRT and has thus demonstrated the pre-clinical suppression of growth and induction of apoptosis [43]. By specifically blocking this complex, the pharmacological profile would be potentially more favorable, as would its therapeutic window [43]. Interestingly, experiments by Anastas et al. on DIPG cells and xenografts showed an inhibition of growth by inducing differentiation and cell cycle arrest [44]. It also reached sufficient concentrations in brain tumors when delivered via convection-enhanced delivery (CED).

For their part, LSD1/KDM1A inhibitors are still in pre-clinical studies for glioblastoma/glioma and medulloblastoma [45,46,47], while they are advancing into the early phase for other clinical indications (hematologic malignancy and solid tumors) [48].

## 4. IDH Inhibitors (Vorasidenib, Ivosidenib)

The well-known IDH mutation molecularly defines a subgroup of CNS tumors, astrocytomas grade 2–4, and co-deleted 1p/19q oligodendrogliomas. These groups of IDH-mutant gliomas have a notably better prognosis than IDH wild-type gliomas [49]. Unfortunately, most of them will relapse, and new therapies are needed to fill this unmet clinical need. Although considered rare in pediatrics, in a recent genomic study of 50 adolescents and young adults (AYAs) with HGGs and high-risk CNS tumors, 50% were found to be IDH-mutant, either diagnosed upfront or identified/reclassified by methylation analysis [50].

IDH mutations affect proteins catalyzing the reversible oxidative decarboxylation of isocitrate to alpha-ketoglutarate (aKG) while reducing NADP^+^ to NADPH. As a result, the mutant enzymes convert NADPH and aKG into NADP^+^ and D-2-hydroxyglutarate (D-2HG), the latter of which accumulates in large quantities in IDH-mutant cells. Secondly, D-2HG competitively inhibits aKG-dependent dioxygenases, which are involved in numerous cellular processes (response to hypoxia, epigenetics, angiogenesis, etc.) and subsequently drives gliomagenesis [51,52]. 

Vorasidenib is a dual inhibitor of IDH1 and IDH2 mutant proteins and has the ability to penetrate the blood–brain barrier. It was approved by the FDA in August 2024 for patients aged 12 and over with grade 2 astrocytomas or oligodendrogliomas with IDH1 or IDH2 mutation post-surgery. Ivosidenib, meanwhile, is an IDH1-mutant inhibitor. It has been studied in adult patients with grades 2–4 IDH-mutant gliomas with around two-thirds of patients having stable disease [53].

The safety profile of both molecules is favorable, with transaminase elevations being the most common grade 3+ side effects and QT prolongation at high doses [54,55].

In order to select a molecule for phase III, Mellinghoff and colleagues led the randomized perioperative phase I of vorasidenib against ivosidenib. This study has decided in favor of vorasidenib for the INDIGO study due to its better 2-HG suppression and its brain penetrance [56]. No children were included in this study.

The extent of resection in patients with IDH-mutant gliomas correlates with better outcomes [57]. Generally speaking and based on the latest data, patients with grade 4 astrocytomas benefit from adjuvant irradiation and temozolomide. Similarly, patients with IDH-mutant grade 3 glioma undergo radiotherapy followed by either TMZ or procarbazine/CCNU/vincristine (PCV) [58,59]. For grade 2 patients, a personalized approach is required, as no consensus exists.

However, the international phase III INDIGO study evaluating vorasidenib versus placebo for patients 12 years or older with grade 2 non-enhancing gliomas demonstrated a PFS benefit in patients with a measurable residual tumor not requiring radiotherapy or chemotherapy in favor of vorasidenib [60]. Indeed, the vorasidenib group had a PFS of 27.7 months, while the placebo group had a PFS of 11.1 months. The time to next surgery was also longer, as was tumor growth. However, crossover was permitted, and several patients had residual lesions of more than 2 cm, suggesting higher-risk characteristics. 

Further studies are therefore needed to clarify the long-term effect, optimal timing, and combination required (Table 1). Also, studies for pediatric patients and those under 12 years of age are needed. As early radiotherapy is not generally favored in pediatrics, if vorasidenib proves an effective option, this would be a promising step forward.

Other IDH inhibitors are under development, as reviewed by Baek et al. [61].

## 5. CDK Inhibitors (Ribociclib, Palbociclib, Abemaciclib)

The CDKN2A locus encodes the tumor suppressors p16(INK4A) and p14(ARF). P16 is an inhibitory protein kinase, playing a central role in restricting cyclin-dependent kinase (CDK) 4/6 activity to keep cells in the G1 phase of the cell cycle. In the absence of p16, elevated CDK4/6 activity manifests itself in hyperphosphorylation of the downstream cycle and the retinoblastoma protein (RB), which in turn promotes cell replication via advancement through the G1/S phase.

The homozygous deletion of tumor repressor CDKN2A/B emerges frequently at the recurrence of IDH-mutant glioma, driving poor patient outcomes [62,63]. Nasser and colleagues investigated the effect of palbociclib and abemaciclib in IDH-mutant patient-derived cell models and orthotopic xenograft tumor models. When CDKN2A is inactivated in these models, CDKi administration improves their survival [64]. Palbociclib was evaluated in a phase I clinical trial PBTC-042, which included children and young adults with refractory CNS tumor; however, no patient had an objective response to the therapy [65]. 

Ribociclib (LEE001) was combined with everolimus in phase I in a refractory brain tumor population [66]. RP2D had been established in pediatrics at 350 mg/m^2^ 21d/28, with mainly myelosuppression as a side effect [67]. CNS penetrance was also reported to be adequate [34,66]. A phase I/II trial was also conducted in 10 newly diagnosed DMG/DIPG patients post-radiation [68]. The median OS was 16.1 months, and increased necrotic volume was observed on four MRIs after two evaluations. A phase II trial in combination with everolimus is currently ongoing for patients with HGGs and DIPGs (Table 1).

In in vivo and in PDX models of high-grade H3G34 gliomas, Liu and colleagues demonstrated that CDK6 inhibition by ribociclib acts on interneuron lineage progenitor-like states [69]. Promisingly, this finding was then demonstrated in a 10-year-old girl with diffuse hemispheric glioma (DHG)-H3G34-mutant in second relapse, in whom ribociclib stabilized the disease for 18 months [69].

Medulloblastomas have an intact retinoblastoma protein (RB), suggesting that CDK4/6 inhibition might be effective [70]. In addition, CDK2 inhibition was found to be synthetically lethal to MYCN-driven overexpressing cancer cells [71]. So, in order to simultaneously target MYC, mTOR/PI3K, and cyclin pathways, a combination of ribociclib was tested with BET-bromodomain JQ1 and paxalisib on group 3 medulloblastoma cell lines [72]. In vivo, ribociclib showed single-agent activity in medulloblastoma models despite in vitro synergy with paxalisib, while JQ1 lacked activity in vivo. Another team has also combined JQ1 and milciclib and found that they synergistically reduced MYC-driven medulloblastoma cell survival and prolonged survival in allografted mice [73].

## 6. Selinexor (KPT-330)

Selinexor is a brain penetrant oral inhibitor of XPO1, a nuclear transport protein with high BBB penetrance. Notably, it is the sole nuclear exporter of tumor suppressors, including p53, Rb, and p27 [74]. XPO1 is overexpressed in many cancers, including high-grade gliomas (HGGs) [75] and DIPGs [76], leading to the mislocalization of these proteins outside the nucleus. Thus, selinexor has the potential to restore TP53 function, among other effects.

Pre-clinical studies demonstrate efficacy in DIPG/DMG and other pediatric HGG models [76]. Synergy was also found with radiotherapy in experiments by Wahba et al. [77]. Orthotopic xenograft models of GBM treated with selinexor and radiotherapy survived significantly longer than those treated with selinexor alone or radiotherapy alone. This drug was studied in phase II trials in adults with recurrent glioblastoma (KING trial, ORR 10%) and in phase I pediatric trials with recurrent solid and brain tumors (ADVL1414), which established an RP2D at 35 mg/m^2^/dose per week [78,79]. The main side effects were fatigue, increased liver enzymes, acute reversible neurologic changes, and myelosuppression.

The COG is currently investigating the efficacy of selinexor in the pediatric population with de novo DIPG/DMG and HGG (ACNS1821) concomitantly and adjuvantly with radiotherapy (Table 1). Unfortunately, interim analysis revealed that selinexor did not lead to improved outcomes in patients with DIPG, and this stratum was closed to further accrual; patients with DMG receiving selinexor were also recommended to discontinue protocol therapy. However, the stratum for patients with hemispheric HGG will likely reopen for accrual, and those patients studied can continue this therapy.

## 7. TP53-Targeted Therapies

The TP53 mutation, known as the guardian of the genome, is found in a large proportion of brain tumors and is associated with poor prognosis, as well as chemo- and radio-resistance. In particular, it is found in 90–95% of patients with H3.3-G34-mutant gliomas and 53% of H3K27M [13,80]. 

It would seem straightforward that restoring its function would allow it to act as an anti-cancer therapy, but there are still many clinical translations lacking in filling this gap, particularly for patients with brain tumors.

APR-246 is a compound that can restore wild-type (wt) TP53 function in mutant TP53 proteins. It is reported to convert in cells into a reactive electrophile methylene quinuclidinone (MQ). MQ then reacts with cysteine residues C124, C229, and C277 in the core of mutant TP53 proteins. The latter reaction thereby changes their conformation from “mutant” to “wt”, resulting in the restoration of the transactivation of wt TP53 target genes that inhibit tumor growth [81]. This MQ can influence a very large number of proteins, including several enzymes, and could therefore probably act more on a wider scale [82,83]. Interestingly, Wang and colleagues demonstrated that APR-246 also acts on TP53wt malignant cells by activating them via the induction of several programmed cell death pathways, including apoptosis, necroptosis, and ferroptosis [84].

Also, in study by Michaeli et al., APR-246 treatment enhanced sensitivity to alpha radiation, leading to reduced tumor growth and increased rates of tumor eradication, which is interesting for the treatment of several entities requiring radiation and mutated TP53 [85].

Unfortunately, this agent has not yet been studied in children. Phase 1B results in combination with pembrolizumab concerned non-CNS tumors with an acceptable toxicity profile [86].

## 8. Aurora Kinase A Inhibitors (MLN8237, Alisertib)

The Aurora kinases are a family of enzymes that control several aspects of cell division. Their dysfunction or dysregulation has been associated with aneuploidy/tetrapoloidy and tumorigenesis [87]. Aurora kinase A (encoded by the gene *AURKA*) regulates centrosome formation, stability, and mitosis. It is frequently overexpressed in many cancers, including breast, lung, and head/neck cancer [88].

The INI1 tumor suppressor complex normally represses Aurora A transcription; therefore, INI1 loss, as characteristically seen in rhabdoid tumors, leads to high expression of the kinase [89]. This relationship has led to an investigation of Aurora kinase A inhibitors as a possible therapeutic option for patients with atypical teratoid rhabdoid tumors (ATRTs).

One of the earliest reports of MLN8237 (Alisertib) use in ATRTs was from St. Jude Children’s Research Hospital, where four patients with recurrent/refractory disease were treated at the recommended pediatric phase II dose of 80 mg/m^2^ once daily on days 1–7 of a 21-day cycle [90,91]. All patients were reported to have had disease stabilization or regression after 1–2 cycles, with a median duration of stable disease of 11.0 months.

This led to SJATRT, a phase II trial in patients aged <22 years with recurrent ATRTs [92]. Among 30 evaluable patients, only one PR and eight SD were achieved at 12 weeks of treatment, with 6-month and 1-year PFS estimates of 30.0% and 13.3%, respectively. Treatment was generally well-tolerated, with the most common grade 3/4 toxicities being neutropenia (77%), anemia (33%), lymphopenia (27%), thrombocytopenia (27%), and febrile neutropenia (23%). All patients alive at the time of analysis went on to receive other salvage therapies. Also, the phase II study led by the COG (ADVL0921) using monotherapy for recurrent/refractory tumors had a general ORR of less than 5%, with no response among the four patients with rhabdoid tumors [91]. This is in contrast to a case report of a single patient with recurrent ATRTs who achieved remission after 8 months of therapy, was continued on treatment for a total of 4 years, and continued to be in remission 3 years after stopping therapy [93].

In a different vein, AURKA is also known to increase the half-life of c-MYC and N-MYC oncoproteins by protecting them from proteasome-dependent degradation [94]. Therefore, AURKA inhibitors have been shown to destabilize MYC-family oncoproteins and to have an anti-tumor effect in vivo [95,96]. Chang and colleagues have recently tested a new long half-life AURKA inhibitor (DBPR728) in MYC-overexpressing medulloblastoma xenografts [97]. The latter molecule was also found to be synergistic with the mTOR inhibitor everolimus, while AURKA inhibitor AT9283 was also potentiated with dasatinib pre-clinically [98].

## 9. Brigatinib (Tyrosine Kinase Inhibitor)

Brigatinib is a tyrosine kinase inhibitor with activity against several kinases including ALK (anaplastic lymphoma kinase), ROS1, insulin-like growth factor-1 receptor, and epidermal growth factor receptor [99]. Alterations in the ALK gene, most often fusions, drive cell growth and proliferation and have been identified in a number of cancers, including anaplastic large cell lymphoma, non-small-cell lung carcinoma (NSCLC), and neuroblastoma [100]. Initially developed to combat resistance associated with first- and second-generation ALK inhibitors, brigatinib has shown superior efficacy and tolerability in a recent phase III study of adults with NSCLC [101].

NF-2 related schwannomatosis (NF2-SWN, formerly neurofibromatosis type 2) is an autosomal-dominant tumor-suppressor syndrome characterized by a predisposition to the development of multiple tumor types, including benign nerve sheath tumors (schwannomas), meningiomas, ependymomas, and retinal hamartomas, to name but a few [102]. Schwannomas occur most often bilaterally in the vestibular nerves and lead to progressive hearing loss, but they can also be present on other cranial, spinal, or peripheral nerves. In a preclinical study utilizing high-throughput screens and in vivo modeling, it was found that brigatinib inhibited NF2-deficient meningioma and schwannoma cell lines and mouse models [103]. However, neither schwannoma or meningioma cells frankly express ALK. It would seem that brigatinib works on these entities via its action on multiple other tyrosine kinases such as Aurora kinase A (AURKA), cyclin G-associated kinase (GAK), activated Cdc42-associated kinase (ACK1), Fps/Fes-related kinase (Fer), and FAK1 (PTK2) [103]. Of note, focal adhesion kinase (FAK), among others, has previously been recognized as an oncoprotein in NF2-related tumors, as reviewed by Ammoun and Hanemann [104]. Brigatinib was therefore chosen for further study through the Innovative Trial for Understanding the Impact of Targeted Therapies in NF2 (INTUITT-NF2) platform trial.

This trial was a phase II, prospective, multicenter trial that enrolled patients aged 12 years and older with NF2-SWN and the progression of an NF2-SWN-related tumor [105]. A total of 40 patients (with target tumor vestibular schwannomas [10], non-vestibular schwannomas [8], meningiomas [20], and ependymomas [2]) received daily oral brigatinib, including 30% of patients less than 21 years old. The percentage of target tumors with an objective radiographic response (decrease of at least 20% in tumor volume from baseline) was only 10%; however, they exceeded the historical control of 2%. In addition, the projected mean annualized growth rate decreased from 65% before treatment to 8% during treatment. No effect was observed among ependymoma, and 15% (3/20) of meningioma had a radiographic response.

For vestibular schwannoma specifically, radiographic responses were seen in approximately 23% of patients; however, an improvement in hearing intelligibility occurred in 35% of ears, while 27% of ears displayed a decrease. The percentage of patients who had no decrease in hearing at 12 months was 89% for hearing sensitivity and 74% for hearing intelligibility, suggesting a possible benefit of delaying hearing loss.

Treatment with brigatinib was overall safe and well-tolerated, with side effects managed with dose reductions or delays; only one patient experienced an adverse event leading to a discontinuation of therapy.

In a recent retrospective report of brigatinib in 12 patients with NF2 (ages 14–32 years), a significant decrease was seen in the average size of meningioma tumors (9/12 patients) in contrast to little change in vestibular schwannomas (all patients) without significant improvement in hearing [106]. Pain (measured by the visual analog scale) and emotional state (measured by Symptom Checklist-90) scores were significantly lower at 6 and 12 months compared to the baseline.

## 10. Conclusions

While various small-molecule therapies have resulted in promising pre-clinical and early clinical data, many have yet to clearly establish their roles in the treatment of pediatric central nervous system malignancies. Their targeted effects, administration schedules, and generally favorable side effect profiles are desirable, though ultimately, their effectiveness must be demonstrated through larger scale prospective studies. This is a challenge in its own given the relative rarity of many of these diagnoses. Even for those with anecdotal or early trial data, access outside of clinical trials remains limited, with availability restricted to select compassionate use programs. Further research is needed to identify those small molecules with the greatest clinical impact and bring these potentially disease-modifying therapies into mainstream care.

## Figures and Tables

**Table 1:** Small molecules in pediatric neuro-oncology.

Compound	Mechanism of Action/Target	Evaluated Neuro-Oncology Indications	Ongoing Pediatric Clinical Trials (Non-Exhaustive)
Dordaviprone, Imipridone (ONC201, TIC10, NSC350625)	DRD2 antagonist	H3K27M gliomas, DMG, DIPG, glioblastoma	BIOMEDE 2 (NCT05476939); PNOC022 (NCT05009992); ACTION (NCT05580562)
Tazemetostat (EPZ-6438)	EZH2 inhibitor	ATRT, tumors with loss of SMARCB1/SMARCA4 or EZH2 mutations	NCT05228158; NCT05407441
Panobinostat (LBH589)	HDAC inhibitor	ATRT, DIPG, medulloblastoma	NCT04804709
Vorasidenib (S095032/AG-881)	IDH1/2 inhibitors	IDH-mutant glioma	≥12 y.o; VICTORIA (NCT06969352); NCT06780930; NCT06478212 INDIGO (NCT04164901)
Ivosidenib (AG-120)	IDH1 inhibitor		
Ribociclib (LEE01)	CDK4/6 inhibitors	Phase I refractory CNS tumor; phase I/II DMG/DIPG	NCT05843253
Palbociclib			N/A
Abemaciclib			NCT02644460 (completed, no results); NCT06413706
Selinexor (KPT-330)	XPO1 inhibitor	Refractory CNS tumor (Phase I)	ACNS1821 (NCT05099003)
Eprenetapopt (APR-246)	TP53-targeted therapies	None (adult non-CNS tumor)	None
Alisertib (MLN8237)	Aurora kinase A inhibitors	ATRT (Phase II), Adults HGG	NCT02114229
Brigatinib	Tyrosine kinase inhibitor	NF-2-related schwannomatosis-associated tumors	NCT04925609
